# Age and sex related change in tooth enamel thickness of maxillary incisors measured by cone beam computed tomography

**DOI:** 10.1186/s12903-023-03639-y

**Published:** 2023-12-06

**Authors:** Abdulsalam Rashid Al-Zahawi, Rawa Omar Ibrahim, Ranjdar Mahmood Talabani, Shilan Nawzad Dawood, Didar Sadiq Hama Garib, Ako Omer Abdalla

**Affiliations:** 1 Conservative Department, College of Dentistry, University of Sulaimani, Sulaymaniyah, Iraq; 2 Sulaymaniyah General Directorate of Health, Sulaymaniyah, Iraq

**Keywords:** Enamel thickness, Age, Sex, CBCT

## Abstract

**Background:**

To measure adequate enamel thickness of maxillary incisors in planning enamel reduction for a porcelain laminate veneer restoration in relation to chronological age and sex by using cone beam computed tomography (CBCT) in an Iraqi subpopulation.

**Methods:**

From 81 CBCT images, 324 maxillary incisors were examined. Enamel thickness was measured at both mesial and distal regions of the tooth in three different levels: cervical, middle, and incisal (occlusal) 1/3 at a sagittal section. Measurements were made for the following tooth areas using CBCT: facial enamel thickness at 1, 3, and 5 mm from the cementoenamel junction (CEJ), palatal enamel thickness at 5 mm from the CEJ (5 mm P), facial and palatal enamel thickness at the incisal edge (IFP), mid incisal enamel thickness (IET), and the incisal edge enamel-pulp distance (IEPD). Relationships of enamel thickness with age and sex were evaluated using Independent t-test, Mann–Whitney U-test and the Pearson correlation coefficient, a simple linear regression analysis used for statistical analysis.

**Results:**

Significant differences (*P* < 0.05) were found in terms of an inverse association between enamel thickness and chronological age at all measurements above the CEJ and the regression model for the mid-incisal enamel thickness was (R2 of 0.4). In contrast, there was an increase in IFP, palatal, and IPED enamel thickness with age. Also, significant differences were found in enamel thickness between males and females, the enamel being thicker in females in relation to facial enamel thickness, enamel palatal thickness above CEJ and IET, while for IEPD, the enamel thickness was greater in males compared to females.

**Conclusion:**

The measurements for enamel thickness outcome variables in relation to chronological age revealed significant differences for each measured distance and there were statistically significant differences in enamel thickness between males and females at all measurements except at IFP. These results demonstrate that CBCT can be used for noninvasive, accurate measurements of enamel thickness in both sex.

## Background

Tooth enamel thickness plays a significant role in the bonding of the restoration to the tooth structures, strength of the restoration, tooth color, and biological tooth condition [1, 2]. However, the dentist’s knowledge is a significant factor in planning the amount of tooth reduction for a porcelain laminate veneer (PLV) restoration. With the introduction of resin-bonded ceramics and advances in the production of adhesive systems, cosmetic dentistry with minimal preparation design has become a viable treatment approach.

Color stability, adhesion durability to enamel, and meeting patients’ esthetic demands with less trauma are the major advantages of resin bonded ceramics. An optimal bonded veneer restoration can be obtained, especially if the preparation is located completely in the enamel, as long as accurate adhesive treatment techniques are carried out and suitable resin cement is selected [2–4]. It is essential to limit tooth preparation within the enamel and provide adherence of the minimally invasive resin-bonded ceramic restoration to enamel to avoid failure [3]. It is necessary to consider preparation within the enamel vs no preparation in order to increase the fracture resistance of the veneered restorations [1]. Increased enamel thickness increases the failure load necessary to cause catastrophic failure in the porcelain veneer [4]. Porcelain bonded to enamel has shown much higher fracture strength than porcelain bonded to dentin. The 0.5-mm thick porcelain bonded to enamel has demonstrated higher fracture strength than 1.0-mm thick porcelain bonded to dentin [5]. Enamel thickness is a statistically significant predictor of tooth color change. Enamel thickness has an impact on tooth color, which may inspire researchers and dentists to create restorative materials that closely resemble the color of natural teeth while preserving as much of the current enamel as possible [6]. The enamel hardness (H) and Young’s modulus (E) at the enamel surface are H > 6 GPa and E > 115 GPa, while these measurements change at the enamel-dentin junction to H < 3 GPa and E < 70 GPa. These changes correspond to changes in the prism alignment and chemistry that relate to enamel depth [7].

Available data on the dimensions of the tooth enamel structure, as well as their relationship with the aging process, is limited [8]. Accurate enamel thickness measurement before tooth preparation can reduce the risk of over preparation of the dentin. Different methods are used to measure the enamel or dentin thickness: physical sections, lateral flat plane radiographs method, Radiographic grid method, Digital radiographic technique superimposed with radiolucent grid lines, computer-generated mCT sections, Optical coherence tomography (OCT), and Cone Beam Computed Tomography (CBCT) [8–10]. The lateral flat plane radiograph method is considered inadequate for measuring enamel thickness accurately [11]. CBCT is a new medical imaging technique introduced that generates 3-D images at a lower cost and absorbed dose compared with conventional computed tomography (CT). CBCT has the potential to be an accurate, non-invasive procedure [12]. Although dental measurements from CBCT volumes provide quantitative analysis with a small systematic error, an adjustment for this error allows for improved accuracy [13].

To the authors’ knowledge, one study used CBCT to assess the relationship between enamel thickness and age, but did not take into account how sex would affect that relationship [14]. Therefore, the aim of this study is to measure adequate enamel thickness of maxillary incisors in relation to chronological age and sex by using cone beam computed tomography (CBCT) in an Iraqi subpopulation.

## Methods

### Study design

This study was designed as a retrospective study and carried out at a single Private Oral Radiology Center in Sulaimani city Kurdistan Region/Iraq in the period from 2/20/2021 to 2/1/2022. The study was approved by the Ethics Committee of the University Of Sulaimani College Of Dentistry (No. 152/23; date 29.3.2023).

### Study sample

Three hundred and twenty four maxillary incisor teeth (numbers 11,12,21,22) (132 male and 192 female) from 81 CBCT images were selected from 950 CBCT images. All of the selected sample satisfied the following inclusion criteria: individual aged over 16 years and with all maxillary incisors present, sound teeth, without caries, without root canal therapy or fracture and fully erupted. The exclusion criteria included the following: current orthodontic treatment, prosthetic crowns of maxillary incisors, inflammatory periapical lesions, supernumerary teeth, foreign bodies or previous surgery of the anterior maxilla and inferior technical quality of the scans (incorrect exposure settings, low image resolution, motion artefacts and incomplete coverage of the maxillary incisors).

### CBCT measurements

All CBCT images were acquired with a Carestream 9600 (CARESTREAM DENTAL, FRANCE). Technical specifications were as follows: 10 cm spherical imaging volume, 75μmx5μmx5μmx isotropic voxel size, and a field of view of 10 cm diameter. The CBCT radiographs were taken according to the following parameters: 120 kV, 8 mA, and exposure time of 20 s by CS 3D imaging software 1.9 (Carestream Dental). The CBCT images were examined using the built-in software package in an axial plane. If needed, image contrast and brightness were adjusted for optimal visualization.

**Fig. 1:**
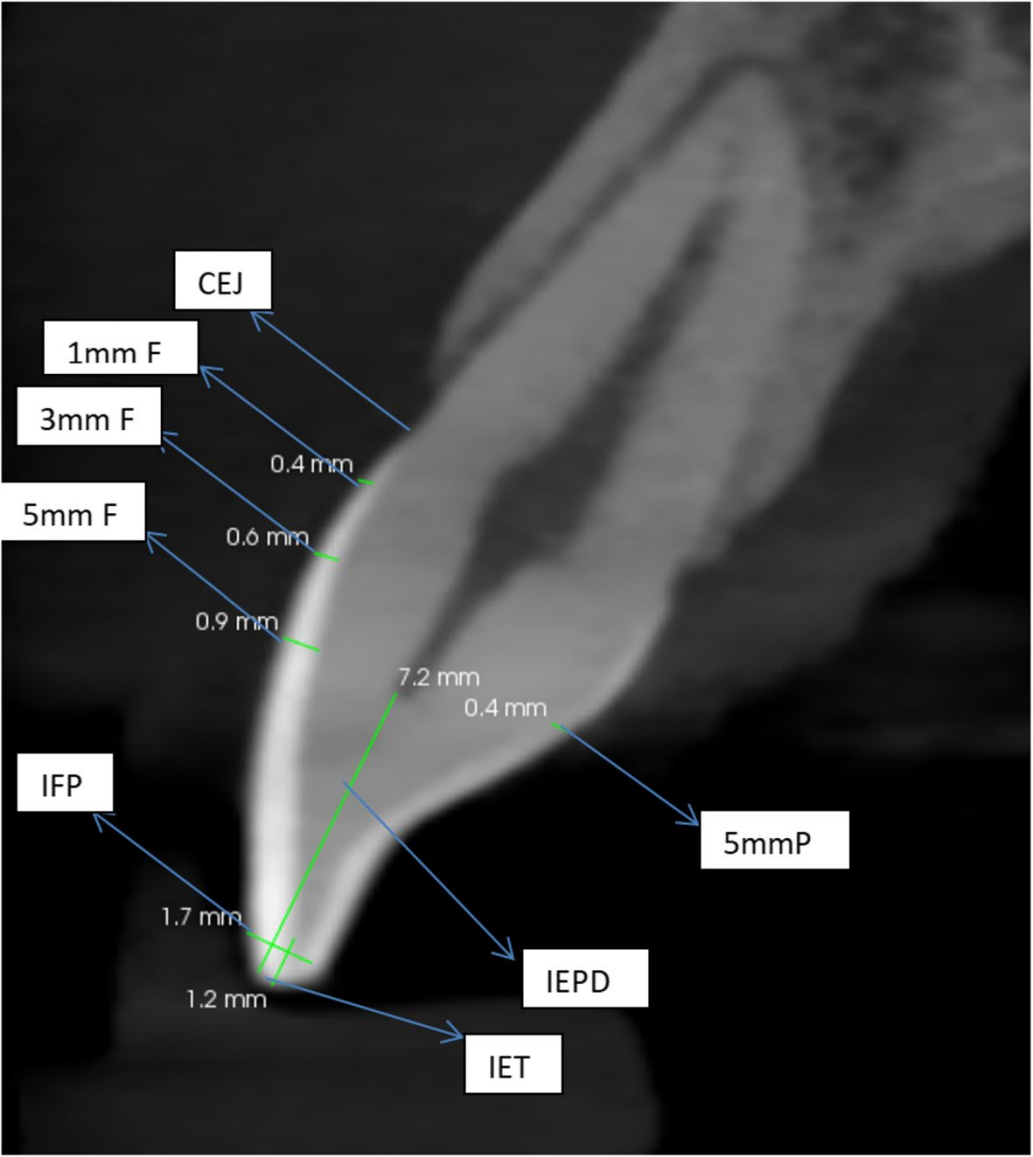
Sagittal section of the maxillary central incisor with tooth landmarks used for enamel thickness measurement

Sagittal profile section of the midline was selected for each tooth and 7 points were selected for measuring the enamel thickness as shown in (Table 1 and Fig. 1).

**Table 1:** Reference points for enamel thickness measurement and their abbreviations

Point name	Abbreviation of point name
Cement-enamel junction	CEJ
1 mm distance from CEJ facial surface	1mm F
3 mm distance from facial surface	3mm F
5 mm distance from facial surface	5mm F
facial and palatal enamel thickness at the incisal edge	IFP
Mid incisal enamel thickness	IET
Incisal enamel pulpal distance	IEPD
5 mm distance from CEJ palatal surface	5mm P

### The standard consistency test (Kappa Value)

All the images were evaluated by two observers retrospectively. At the same time as the reliability test, a routine consistency check (kappa value) of the results was performed. Reliability was rated unqualified when the kappa value was 0.4, moderate when the kappa value was between 0.41 and 0.6, excellent when the kappa value was between 0.61 and 0.8, and totally dependable when the kappa value was between 0.81 and 1.0 [15].

### Statistical analysis

The data were summarized by the use of descriptive statistics. To evaluate the relationship between thickness and age, a quadratic regression model was used. Shapiro–Wilk test was used to determine the normality distribution of the samples regarding the sex and type of tooth. Independent t-test was used for normally distributed data, and Mann–Whitney u test was used for the data that were not normally distributed. Two-way ANOVA was used to assess the interaction between age and sex. Statistical analyses were done with statistical software (SPSS 26.0; SPSS Inc, Chicago, Ill).

## Results

The score from analysis of inter-examiner reliability was 0.87, indicating total dependability of the study. Descriptive statistics of the measurements are presented in (Table 2).

**Table 2:** Descriptive statistics of enamel thickness (mm)

Outcome variable	Numbers of samples (N)	Minimum	Maximum	Mean ± SD
1mm F	324	0.30	1.10	0.58 ± 0.18
3mm F	324	0.12	1.40	0.79 ± 0.21
5mm F	324	0.30	1.90	0.99 ± 0.2
IET	324	0.00	2.30	1.03 ± 0.4
IEPD	324	0.80	9.80	5.2 ± 1.06
5mm P	324	0.20	1.40	0.71 ± 0.2
IFP	324	0.90	3.30	1.9 ± 0.4

The age of the samples was ranged from (14 to 68) years old with a mean age of (35.6 ± 12).

The hypothesis tests if age carries a significant impact on enamel thickness. The results were not normally distributed according to Shapiro Wilk statistic test; therefore, a quadratic regression was used to follow the negative skewness and kurtosis of the data. The dependent variable (age) was regressed on predicting variable(enamel thickness on the selected points) to test the hypothesis H_1_.Age significantly predicted enamel thickness on point(1mm F) F(2,321) = 21.763, *p* < 0.001.The beta coefficient is positive in age and negative in age^2^, which indicate that increase in age lead to decrease in enamel thickness on point (1mmF) (inverse relationship).While the *R*^*2*^ = 0.143 depicts that the model explains 14.3% of the variance in point(1mmF). Table 3 shows the summery of the finding.

**Table 3:** Results of regression analysis

Dependent variable	Predictor	R^2^	Coefficient	Standard error	ANOVA	
					**F**	**P**
1mm F	Age	0.143	0.256	21.3	21.763	< 0.001
	Age^2^		-0.627	16.66		
3mm F	Age	0.167	0.33	15.4	32.158	< 0.001
	Age^2^		-0.733	9.3		
5mm F	Age	0.117	0.23	15.3	21.31	< 0.001
	Age^2^		-0.56	7.4		
IET	Age	0.47	0.19	5.72	9.02	< 0.001
	Age^2^		-0.40	2.58		
IEPD	Age	0.048	-0.59	3.28	9.21	< 0.001
	Age^2^		0.789	0.29		
5mm P	Age	0.073	-0.83	17.3	12.65	< 0.001
	Age^2^		0.63	11.3		
IFP	Age	0.021	-0.72	9.79	3.47	0.032
	Age^2^		0.618	2.46		

*P* < 0.05 is significant; R^2^_:_ coefficient of determination

The regression analysis revealed significant differences (*P* < 0.05) in all of the relationships between enamel thicknesses and age (Table 3). Outcome variables of enamel thickness in relation to age were represented as coefficients of determination (R^2^).

**Fig. 2:**
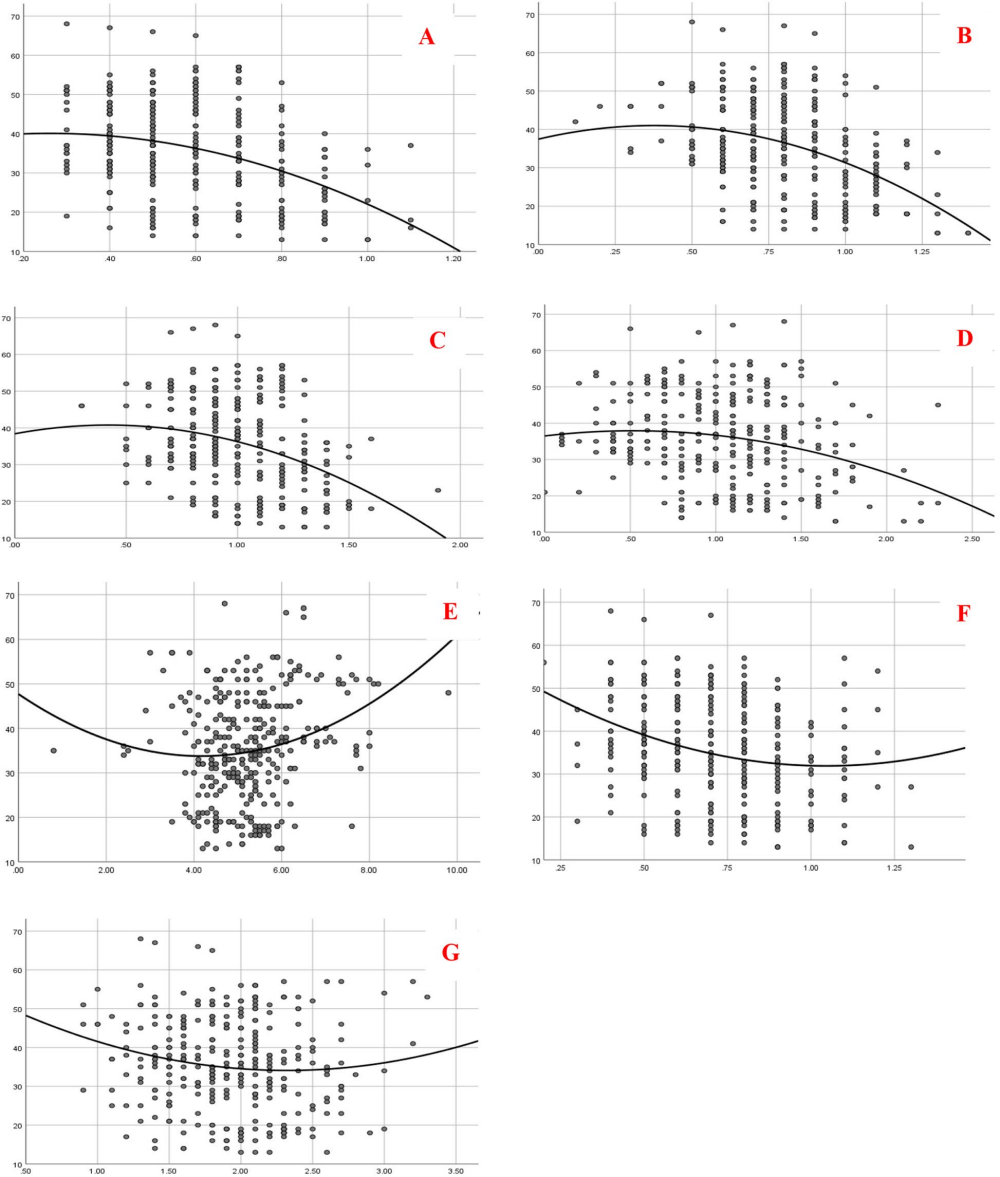
Scatter plots for actual measurements and predicted values (line of best fit) of enamel tooth thickness (horizontal axis) versus age (vertical axis); **A** 1 mm F; **B** 3 mm F; **C** 5 mm F; **D**: IET; **E** IEPD; **F** 5 mm P and **G** IFP

The results of the analysis revealed an inverse relationship between enamel thickness and chronological age at (1mm F, 3mm F, and 5mm F, respectively), with the same finding obtained for IET, which was the best fit in the regression model (R^2^ of 0.4) (Fig. 2 A, B, C, D). Meanwhile, an increase was observed in the IEPD, 5mm P, and IFP in relation to age (Fig. 2 E, F, G).

According to the Shapiro–Wilk normality test; only the IFP group was normally distributed; therefore, the independent t-test was used. Since the other groups were not normally distributed, the Mann–Whitney u test was used for these groups.

Regarding facial-enamel thickness, at 1,3,5 mm above CEJ, 5mm P, and IET, there was a statistically significant difference in enamel thickness between males and females, with thicker enamel found in females (Table 4).

**Table 4:** Differences in outcome variables in relation to sex

Outcome variable	Sex	N	Mean ± SD	Median	Interquartile range	*P* value*
1mm F	male	132	0.54 ± 0.16	0.5	0.2	< 0.001
	female	192	0.62 ± 0.17	0.6	0.2	
3mm F	male	132	0.72 ± 0.18	0.7	0.3	< 0.001
	female	192	0.83 ± 0.22	0.8	0.3	
5mm F	male	132	0.91 ± 0.2	0.9	0.2	< 0.001
	female	192	1.04 ± 0.25	1	0.3	
IET	male	132	0.97 ± 0.42	0.95	0.57	0.011
	female	192	1.06 ± 0.4	1.1	0.40	
IEPD	male	132	5.64 ± 1.1	5.5	1.30	< 0.001
	female	192	4.95 ± 0.9	4.9	1.07	
5mm P	male	132	0.66 ± 0.18	0.6	0.3	< 0.001
	female	192	0.74 ± 0.2	0.8	0.3	
IFP	male	132	1.86 ± 0.3	1.8	0.5	0.086*
	female	192	1.95 ± 0.4	2	0.6	

^*^No significant difference at *P* < 0.05

Regarding the IEPD, the distance was greater in males in comparison to females, and this difference was statistically significant. For IFP there was no significant difference between males and females (Table 4).

According to the Shapiro–Wilk normality test, none of the outcome variables were normally distributed; therefore, Mann–Whitney u test was used for all groups. There was a statistically significant difference in facial enamel thickness 5 mm above CEJ, IET, and IFP, with the enamel being thicker in lateral incisors in comparison to central incisors. However, regarding the IEPD the enamel was thinner in lateral incisors compared to central incisors (*P* < 0.001). Regarding the other outcome variables, no significant difference was observed between the central and lateral incisors (Table 5).

**Table 5:** Differences in outcome variables in relation to the type of tooth

Outcome variable	Type of tooth	N	Median	Interquartile range	*P* value*
1mm F	Central incisor	163	0.6	0.2	0.73*
	Lateral incisor	161	0.5	0.3	
3mm F	Central incisor	163	0.8	0.3	0.76*
	Lateral incisor	161	0.8	1.1	
5mm F	Central incisor	163	0.9	0.3	< 0.001
	Lateral incisor	161	1	0.3	
IET	Central incisor	163	1	0.6	0.019
	Lateral incisor	161	1.1	0.5	
IEPD	Central incisor	163	5.4	1.2	< 0.001
	Lateral incisor	161	4.9	1.1	
5mm P	Central incisor	163	0.7	0.3	0.673*
	Lateral incisor	161	0.7	0.2	
IFP	Central incisor	163	1.8	0.6	0.003
	Lateral incisor	161	2	0.6	

^*^No significant difference at *P* < 0.05

Since there were statistically significant differences between the sex, the regression analysis performed separately by sex as seen Table 6.

**Table 6:** The regression analysis of the effect of age on the enamel thickness for both sexes

Dependent variable	Predictor	Male				Female			
		**R**^**2**^	**Coefficient**	**ANOVA**		**R**^**2**^	**Coefficient**	**ANOVA**	
				**F**	**P**			**F**	**P**
1mm F	Age	0.162	0.797	12.94	< 0.001	0.105	0.215	11.12	< 0.001
	Age^2^		-1.177				-0.534		
3mm F	Age	0.126	0.549	9.28	< 0.001	0.144	0.202	15.9	< 0.001
	Age^2^		-0.889				-0.575		
5mm F	Age	0.101	0.86	21.31	< 0.001	0.102	0.29	10.73	< 0.001
	Age^2^		-1.14				-3.28		
IET	Age	0.413	0.221	8.246	< 0.001	0.307	0.47	6.80	< 0.001
	Age^2^		-0.546				-0.65		
IEPD	Age	0.125	0.761	9.21	< 0.001	0.02	-0.673	1.93	0.0148
	Age^2^		-0.415				0.707		
5mm P	Age	0.068	-0.83	12.55	0.001	0.070	-1.335	10.63	0.001
	Age^2^		0.62				1.116		
IFP	Age	0.018	-0.158	3.21	0.0424	0.021	-0.176	3.42	0.034
	Age^2^		0.608				0.614		

Although there are minor changes in the ANOVA(F) values between male and female, but all the results are still significant in relation to age (as seen in the *p* value of the ANOVA test in Table 6). As for betta coefficient, there is a negative relationship between enamel thickness and chronological age at (1 mm F, 3 mm F, and 5 mm F, and IET), Meanwhile, an increase in enamel thickness in relation to age was observed in the point IEPD, 5 mm P, and IFP for both sexes.

To calculate the interaction between age and sex, and to conduct two-way ANOVA, the sample’s age was divided into three age groups: group 1(16–30), group 2(31–50), and group 3(51–68). Significant difference in age-sex interaction at IEPD and IFP groups (Table 7).

**Table 7:** Two-way ANOVA of the transformed data to assess the age-sex interaction

Dependent variable	Age Group		Chronological sex		Age-Chronological sex	
	F	*P* value	F	*P* value	F	*P* value
Facial-enamel at 1 mm above CEJ	23.17	< 0.001	10.13	0.002	2.223	0.110*
Facial-enamel at 3 mm above CEJ	33.77	0.002	7.43	0.07	0.323	0.058*
Facial-enamel at 5 mm above CEJ	18.54	< 0.001	13.21	< 0.001	0.869	0.420*
Incisal enamel thickness (IET)	9.6	< 0.001	4.02	0.02	0.426	0.654*
Incisal enamel-pulp distance (IEPD)	9.72	< 0.001	9.21	< 0.001	5.56	0.004
Palatal-enamel at 5 mm above CEJ	11.25	< 0.001	10.65	< 0.001	1.699	0.184*
Incisal facial-palatal distance (IFP)	3.285	0.045	1.35	0.058*	3.075	0.048

^*^No significant difference at *P* < 0.05

## Discussion

There is limited information on the micrometric enamel thickness for tooth preparations considering age and sex of the study population. Ferrari et al. [16] measured the thickness of enamel using a laboratory caliper of 10 maxillary central incisors, but without considering chronological age. Other studies used SEM to measure enamel thickness in human extracted maxillary central incisors for a population with an age range of 35 to 70 years [8, 17]. Miyagi et al. [18] used optical coherence tomography to verify the precision of enamel thickness measurements of maxillary central and lateral incisors, again without considering sex or age. Pahlevan et al. [18] investigated the thickness of enamel only at the gingival, middle and incisal thirds of the labial surface of extracted maxillary incisors teeth, using stereomicroscopy, without considering age and sex. Belgın et al. [19] examined enamel thicknesses and maximum cervical crown widths of 15 extracted premolar teeth using both Micro-CT and periapical radiographs; Akli et al. [20] measured the enamel thickness of extracted 32 maxillary canine using microcomputed tomography scans, without age and sex characterization; while Feeney et al. [21] employed microtomography to virtually image, section, and quantify the average enamel thickness of a sample of clinically extracted Indonesian canine and premolar teeth in a study that compared males and females.

Only two studies have used CBCT to measure enamel thickness. Brokos et al. [14] examined in vivo the possible variations in enamel thickness among upper anterior teeth using 3D CBCT data, but in only 24 patients aged from 21 to 75 years and without sex consideration, while a study by Salam et al. [22] evaluated enamel thickness of the mandibular canine and mandibular first molar for sexual dimorphism in an Egyptian population sample using CBCT.

To the best of our knowledge, this is the first retrospective study that has correlated age with sex to measure enamel thickness in maxillary incisor teeth using CBCT in planning for minimizing enamel reduction in a porcelain laminate veneer restoration.

In the present study, CBCT was used to measure enamel thickness as it is a nondestructive high resolution three dimensional diagnostic procedure, with rapid scan time and low radiation dose, and is therefore proposed as an alternative method of accurate measuring dental tissue thickness [10, 23].

Because maxillary central incisors are the teeth cited in clinical research as most frequently receiving porcelain laminate veneer restoration [24–26], central and lateral incisors were employed in the current investigation to analyze enamel thickness.

Comparable measurements from earlier studies [8, 17, 24, 27] were taken of the thickness of the facial enamel, and the results showed that, contrary to what other authors have suggested, an enamel reduction of 0.5 mm, which is considered the ideal depth for porcelain laminated veneers, may expose dentin at the cervical area. The enamel layer is thought to have the strongest ceramic bonding to teeth. Clinically, when veneers are only partially adhered to dentin, the likelihood of failure increases. As a result, because tooth reduction in the cervical region usually goes beyond the boundaries of enamel tissue, this has been identified as a problem [16, 28].

Based on the findings from this investigation, increasing the enamel thickness from cervical to incisal (0.58 mm, 0.79 mm, and 0.99 mm, at 1, 3, and 5 mm above CEJ, respectively) and achieving a mean thickness of enamel at incisal edge of 1.03 ± 0.4 requires consideration during tooth reduction if the completed preparation is to remain in the enamel. The results obtained from this study showed thicker enamel in the Iraqi subpopulation at all measurements on the facial surface above CEJ and at incisal edge. Other studies [8, 26, 29] have noted that preparation depth may be in the range of 0.3 to 0.7 mm and 0.79 ± 0.03 at incisal edge and these differences may be related to the method of enamel thickness measurement.

The tooth preparation techniques for PLVs [24–26] have been carried out without consideration of age or sex. In the current study, a regression model was used to predict a tooth's thickness depending on the individual's age. This will allow a clinician to know approximately how much tooth thickness is available for reduction when restoring a tooth for a veneer.

The findings of the current study indicate that with advancing of age, tooth enamel thickness decreased at 1, 3, and 5 mm above the CEJ and IET. This raises the possibility of dentin exposure, which could make the bond less reliable, and these results are comparable with other studies [8, 14]. The mean enamel thickness of the IEPD in the current study utilizing CBCT was 5.2 ± 1.06 and seemed to be comparable to the data previously published for the age range of 30 to 69 years [8, 30].

This study also found that 5 mm P height increased with increasing chronological age. Reduction in the size of the pulp chamber with aging may be the cause of this rise [31]. According to this study's findings, wear and attrition caused the maximal IFP to rise with advancing years. This could be explained by the shape of human incisor teeth, which have a greater cross-section area at the CEJ than the incisal edge. As people age, the thinner incisal section wears down, increasing the maximum incisal-palatal width at the incisal edge.

The results of the current investigation demonstrated that sex is a factor that affects enamel thickness since, with the exception of IEPD, females displayed higher values of enamel thickness than males in all dimensions. A similar finding was observed by Brokos et al. [14] and this may be related to lower masticatory forces in females, which may help to prevent enamel erosion. However, other studies [21, 22] reported that males have significantly greater enamel thickness measurements, dentin area and enamel–dentin junction length than females and these sex differences may be attributed to variations in type of teeth, ethnic population and methods of measurement.

In regard to differences in enamel thickness between maxillary central and lateral incisors, in contrast to the IEPD, which was thinner in lateral incisors compared to central incisors, there was a statistically significant difference in facial enamel thickness 5 mm above CEJ, IET, and IFP, where the enamel was thicker in lateral incisors compared to central incisors (*P* < 0.001). These results align with those reported by Brokos et al. [14], who found that the mean values of enamel thickness for permanent central and lateral incisors were very similar (CI: 734μm, LI: 745μm), which can be explained by the fact that they emerge into the mouth at roughly the same time and have roughly the same length of development time (7–9 years).

To address the limitations of the present study, the use of a larger sample of the Iraqi subpopulation and different types of teeth could lead to a better understanding of how enamel thickness varies in relation to chronological age and sex.

## Conclusions

This study concluded that CBCT scanning allows reliable accurate nondestructive measurement of enamel thickness. It can also be concluded that age and sex both affect enamel thickness; enamel thickness of maxillary incisors decreases with advancing age, while enamel in lateral incisors was found to be thicker in females except in the case of IEPD.

## Data Availability

The data used to support the findings of this study are available from the corresponding author upon request. https://docs.google.com/spreadsheets/d/1uR10M_-AsaKEcam1RVaCp7RPDFt0oTpB/edit?usp=share_link&ouid=115884346681842745188&rtpof=true&sd=true.

## Abbreviations

CBCT: Cone beam computed tomography
CEJ: Cemento-enamel junction
P: Palatal
IFP: Facial and palatal enamel thickness at the incisal edge
IET: Mid incisal enamel thickness
IEPD: Incisal enamel pulpal distance
